# Full evaluation of the psychometric properties of COPSOQ II. One-year longitudinal study on Polish human service staff

**DOI:** 10.1371/journal.pone.0262266

**Published:** 2022-01-26

**Authors:** Łukasz Baka, Monika Prusik, Jan Hyld Pejtersen, Krzysztof Grala

**Affiliations:** 1 Central Institute for Labour Protection–National Research Institute, Warsaw, Poland; 2 University of Warsaw, Warsaw, Poland; 3 VIVE–The Danish Center for Social Science Research, Copenhagen, Denmark; 4 The Maria Grzegorzewska University, Warsaw, Poland; Medical University Innsbruck, AUSTRIA

## Abstract

**Purpose:**

The aim of the study was the full evaluation of the psychometric properties of the COPSOQ II in one-year longitudinal study on human service staff in Poland. Data were collected from 599 employees representing three occupational groups related to human service work.

**Methods:**

CFA was conducted in the structure proposed by the author of the original tool, based on one model, which included 119 observable variables forming 33 latent variables (single item subscales were excluded from analysis). To our knowledge, this was the first complete validation of the entire model using CFA. Reliability analysis was performed using two methods: internal consistency analysis and test-retest analysis. Predictive validity was assessed by correlating COPSOQ II variables with ten criterion variables related to job demands, job resources, work-family conflicts, mental health and well-being.

**Results:**

According to the results, CFA supported the original structure of the COPSOQ II. Most of the 33 subscales were characterized by good or very good psychometric parameters. The obtained results confirmed also the fairly high reliability, as well as high convergence validity of all subscales of COPSOQ II.

**Conclusion:**

The final conclusion is that COPSOQ II is characterised by satisfactory psychometric properties and could be successfully used to fulfil the demand for reliable and comprehensive assessment methods also in Polish job market settings.

## Introduction

Psychosocial hazards are serious risk factors in the work environment. A number of meta-analyses [1–4], prospective studies [5–7] and large national population studies [8–11] reveal that harmful working conditions can become a source of severe physical diseases and mental disorders. According to the European Commission [12], a healthy and safe work environment may contribute to increased work efficiency, general social well-being and the economic development of a country. The European Framework Directive on Safety and Health at Work (89/391 EEC) [13] obliges employers to provide healthy and safe working conditions, including identification and assessment psychosocial risk factors in work environment as well as implementation of preventive actions. One of the most difficult problems for an employer to cope with when diagnosing risk factors is how to measure psychosocial risks.

Leka and Jain [14] made a critical analysis of 37 popular tools for measuring psychosocial hazards in the workplace. Most of these tools have been validated in other countries and also used in international research. A certain limitation is, however, that they are based on classic job stress models [15], which have been developed relatively long ago and do not fully include the current labor market challenges, such as new forms of work organization, technological changes, information overload, multitasking, the need for continuous learning and faster pace of life [16]. Moreover, they were developed from an analysis of industrial work; therefore, they emphasize mainly quantitative workload and disregard the aggravating role of other types of job demands, e.g. emotional demands or demands for hiding emotions [14], crucial for professionals working in human service organizations [17], whose “principal function is to protect, maintain, or enhance the personal well-being of individuals by defining, shaping, or altering their personal attributes” ([18] p. 1).

*The Copenhagen Psychosocial Questionnaire* (*COPSOQ* II, the revised version of COPSOQ) is a measurement tool that covers a wide spectrum of psychosocial work conditions and includes the specificity of the modern labor market and professions [19]. The International Labor Organization [20] and World Health Organization [14] have referred to it as an available measurement tool to evaluate psychosocial hazards in the workplace. In developing COPSOQ II, the authors followed the recommendations formulated for the previous questionnaire version, as well as other tools for studying psychosocial working conditions [19,21]. First of all, they wanted the questionnaire to cover the widest possible number of areas of psychosocial working environments. This is why they did not base the questionnaire on one theoretical model–as is the case for the majority of questionnaires–but referred to several different concepts. The concepts included the ones which Kompier [15] listed as the seven most influential models of occupational stress. COPSOQ II covers a wide range of psychosocial working conditions and can, therefore, be used in all labor market sectors, e.g. industry and social service. Secondly, in developing the questionnaire, the authors took into account recommendations concerning the length of the developed tools and the number of questions for each dimension of psychosocial working conditions [21]. COPSOQ II consists of 41 subscales (related to seven work domains), the majority of which include two to four questions (in total: 127 questions). Thirdly, COPSOQ II contains questions which refer to different levels of human functioning at work (e.g. organization, department, employee) and, as such, analyses can be carried out on different levels of generality, ranging from general demands at work to particular ones (e.g. emotional demands). Moreover, the questionnaire refers not only to potential sources of job stress but also to a human’s own resources (e.g. social support and self-efficacy), as well as mental health (e.g. depression) and well-being at work (e.g. job satisfaction).

### Current study

The COPSOQ has been translated into at least 25 languages and has been validated in a number of countries worldwide [22]. However, none of them fully confirmed the factor structure of the entire instrument, taking into account both the number of subscales and domains. As stated before, COPSOQ II consists of 41 subscales (including a total of 127 items) which are assigned to one of the seven work domains, i.e.: Demands at work; Work organization and job content; Interpersonal relations and leadership; Work individual interface; Values at the workplace; Health and well-being; and Offensive behavior. Therefore, testing its structure in a single model raises technical and computational problems. It is very difficult to test such an expanded model using Confirmatory Factor Analysis (CFA). The review of the literature suggests that other authors have tried to overcome the technical problems in various ways. Some used Exploratory Factor Analysis [23], while others analyzed the tool by means of CFA, but within each of the seven major domains separately [24,25]. Moreover, some previous studies did not include examination of theoretical relevance at all [25,26] or they investigated it with only a few criteria variables, which did not apply to all work domains [27]. The reliability of the tool, in turn, was usually assessed on the basis of set of data collected in cross-sectional studies, using Cronbach’s Alpha measure, not the test-retest method [21,28,29]. The majority of earlier studies on the validation of COPSOQ II tested the short [27–29] or medium [23,26] version of COPSOQ, mainly in the countries of Western (but not Eastern) Europe and in not social-service sectors, e.g. mechanics, food production, cleaning, textiles, garment and trading [29], education, construction, wholesale, manufacturing, financial and insurance [25].

The purpose of the present study is to complex evaluate the psychometric properties of the full version of COPSOQ II in human service institutions, in Poland. This study focused on testing the general structure of the tool using Confirmatory Factor Analysis, based on one model containing 119 observable variables forming 33 latent variables. Eight COPSOQ II subscales measured with a single question were not included in the analysis. It is worth noting that none of the studies the authors came across have extensively tested the original structure of the COPSOQ II in this way. The issue of theoretical validity has been widely covered. The relevance of COPSOQ II variables were tested based on correlations with ten criteria variables representing four areas (i.e.: job demands, job resources, work-life balance and mental health/well-being) that seem to be compatible with the seven work domains of COPSOQ II. The reliability of the COPSOQ II was assessed by means of two methods: one based on internal consistency analysis and the other using the test-retest method.

## Method

### Participants and procedure

The study population (*N* = 599) included human service staff from the following professions: teachers in resocialization centers for children and youth (*n* = 200); care workers in centers for intellectually disabled children and youth with chronic mental illnesses (*n* = 199); medical psychiatric staff for children and youth (*n* = 200). The occupation specificity, involving intensive and direct contact with other people, related to the need to provide different forms of aid such as saving life and health and regular care of people who are ill, have social problems or are in conflict with the law, and formed the selection criterion for the study population groups.

A longitudinal study was carried out, with a one-year interval between the two measurement points (T1 and T2). The study was conducted in the period between September–November 2017 (T1) and September–November 2018 (T2) at the premises of the facilities where the respondents were employed. All participants were treated in accordance with the ethical guidelines of the Helsinki Declaration. Full confidentiality of the data and anonymity were secured. Participants were asked to fill out the questionnaires and seal them in envelopes, which were subsequently collected by research assistants. Out of 1,000 distributed questionnaires, 751 (75%) were completed in the first step of the study (T1) and 599 (60% of the original pool) in the second stage (T2). Finally, 599 subjects were included in the analysis. The analyzed group consisted of 494 women (82.6%) and 105 men (17.4%), between 20 and 70 years of age (*M* = 42.5, *SD* = 9.39). Work experience ranged from 1 to 39 years (*M* = 14.40, *SD* = 9.96). There were no significant differences in the distribution of *age*, *F*(2, 550) = 2.33, *p* = .099, η_p_^2^ = .01, in the three analyzed occupational groups. There were, however, small (judged by the effect size) but significant differences in the length of service, *F*(2, 532) = 6.62, *p* = .001, η_p_^2^ = .02. Care workers (*M* = 14.23, *SD* = 9.36) on average had less seniority in comparison to the medical staff (*M* = 18.26, *SD* = 11.37, *p* = .001).

### Measures

Apart from the subscales of the COPSOQ II, a few other variables were included in the study and used in the criteria analysis. These variables were categorized into four general groups: job demands, job resources, work-life imbalance, and health and well-being.

#### Job demands

Job demands included interpersonal conflicts at work and workload. Interpersonal conflicts relate to the quality of relationships at work and involve burdensome interactions with superiors and colleagues. These can be of varying severity, from minor quarrels to mental struggles [30]. Workload covers the physical and psychological costs incurred by the employee in carrying out tasks, and is usually measured by the number of working hours, the amount of work performed, the number of activities performed per unit of time, and the subjectively assessed physical and mental effort put into work. These variables were measured with two instruments developed by Spector and Jex [30]: Interpersonal Conflicts at Work Scale (ICAWS) and Quantitative Workload Inventory (QWI). The tools consist of four and five items respectively (e.g. “How often do you get into arguments with others at work?”; “How often does your job require you to work very fast?”), with a five-point response scale, ranging from 1 (*less than once a month*) to 5 (*a few times daily*).

#### Job resources

Job resources included social support at work from supervisors and coworkers. In order to assess these variables, a subscale of the Psychosocial Work Conditions was used [31]. This 16-item subscale measures two sources of social support: from supervisors and from co-workers (e.g. “To what extent can you count on your superiors to give you directions on how to resolve a difficult situation?”*)*. All items are scored from 1 (*very limited extent*) to 5 (*very large extent*).

#### Work-life imbalance

This is represented by work-family and family-work conflicts. These are types of role conflict, in which role requirements related to one area of life make it difficult or impossible to fulfill role requirements related to another area of life [32]. These variables were assessed with the Work-Family and Family-Work Conflict Scale [33]. This is a ten-item instrument (e.g. “The demands of my work interfere with my home and family life”; “I have to put off doing things at work because of demands on my time at home”) with a seven-point response scale, ranging from 1 (*strongly disagree*) to 6 (*strongly agree*).

#### Health and well-being

These include three variables–depression, job burnout and job satisfaction. Depression was assessed using the Center for Epidemiological Studies Depression Scale (CES-D: [34]). The CES-D consists of 20 statements, which measure the frequency of depressive symptoms experienced in the past week. The statements refer to depressed mood, feelings of guilt and hopelessness, psychomotor slowdown and sleep disorders (e.g. “I didn’t want to eat; I didn’t have an appetite”). Answers are provided on a four-point scale, from 0 = *rarely or not at all* (*less than one day*) to 3 = *most of the time or all the time* (*five to seven days*). Job burnout was measured with the Oldenburg Burnout Inventory (OLBI: [35]). This 16-item scale consists of two subscales for exhaustion and disengagement from work. Exhaustion is a response to intensive physical, affective, and cognitive strain; it manifests in fatigue, weariness, and a decrease in energy. Disengagement is expressed by distancing oneself from work and by experiencing negative affect towards it [36]. A five-point response scale ranged from 1 (*I completely disagree*) to 5 (*I completely agree*). Job satisfaction was measured with the Job Satisfaction Survey [37]. This is a 36-item scale with a six-point response scale, ranging from 1 (*highly disagree*) to 6 (*highly agree*), that concerns employee attitudes towards the job and different aspects of the job, such as pay, promotion, communication or nature of work (e.g. “There is really too little chance for promotion on my job”).

### Analytical procedures

For each subscale, the following descriptive statistics were calculated at both measurement points: mean (*M*), median (*Me*), standard deviation (*SD*) and the value of standard measurement error (*SE*). Confirmatory Factor Analysis (CFA) was carried out to check the structure of the hypothesized model. A factor-based approach proposed by the authors of the questionnaire’s original version [19] was followed. Eight COPSOQ II subscales measured with a single question (related to: general health, rumors, conflicts at work, disruptive behavior, sexual harassment, threatening and physical violence, and mobbing) were not included in the analysis. Hence, CFA was carried out on 33 subscales based on one model, which included 119 observable variables forming 33 latent variables. Next, reliability analysis was performed using two methods: one based on internal consistency analysis and the other using the test-retest method. An analysis of theoretical validity was calculated by the means of coefficients of COPSOQ II variable correlations with criteria variables and covered interpersonal conflict at work, workload, co-worker and supervisor support, work-family and family-work conflicts, depression, job burnout (including exhaustion and disengagement from work) and job satisfaction. The subscales of COPSOQ II and all criteria variables fell within the scope of the questionnaire. Descriptive statistics and reliability analyses were carried out for the data collected in both measurements. Other analyses were based on the results obtained in the first measurement.

### Ethics

The study was approved by the Regional Ethics and Bioethics Committee of the Cardinal Wyszyński University in Warsaw (KEiB—31/2020 of June 10, 2020) and informed consent was obtained from all individual participants included in the study.

## Results

### Descriptive statistics

Table 1 presents descriptive statistics for the 33 subscales at both measurement points.

**Table 1:** Descriptive statistics for 33 subscales including two measurement points.

	Time point	*N*	*M*	*Me*	*SD*	*SE*
Quantitative demands	1	592	35.80	37.50	17.46	0.72
Quantitative demands	2	592	35.90	37.50	16.90	0.69
Work pace	1	593	54.73	50.00	19.05	0.78
Work pace	2	593	53.72	50.00	18.52	0.76
Cognitive demands	1	593	66.53	68.75	18.23	0.75
Cognitive demands	2	593	63.08	62.50	18.69	0.77
Emotional demands	1	595	70.36	75.00	18.55	0.76
Emotional demands	2	595	67.24	68.75	19.58	0.80
Demands for hiding emotions	1	594	55.51	58.33	21.58	0.89
Demands for hiding emotions	2	594	57.04	58.33	21.19	0.87
Influence	1	592	49.92	50.00	17.28	0.71
Influence	2	592	49.70	50.00	16.00	0.66
Possibilities for development	1	587	65.44	68.75	19.16	0.79
Possibilities for development	2	587	65.27	68.75	19.18	0.79
Variation	1	592	60.24	62.50	21.09	0.87
Variation	2	592	58.02	62.50	20.15	0.83
Meaning of work	1	587	73.36	75.00	17.41	0.72
Meaning of work	2	587	72.65	75.00	17.89	0.74
Commitment to the workplace	1	594	61.52	62.50	17.87	0.73
Commitment to the workplace	2	594	61.24	62.50	17.11	0.70
Predictability	1	586	60.75	62.50	20.43	0.84
Predictability	2	586	62.54	62.50	19.20	0.79
Rewards	1	587	63.44	66.67	21.86	0.90
Rewards	2	587	63.78	66.67	19.89	0.82
Role clarity	1	587	77.52	75.00	16.51	0.68
Role clarity	2	587	76.17	75.00	17.67	0.73
Role conflicts	1	587	39.96	43.75	20.34	0.84
Role conflicts	2	587	40.04	43.75	19.77	0.82
Quality of leadership	1	571	61.93	62.50	24.45	1.02
Quality of leadership	2	571	60.76	62.50	24.11	1.01
Social support from colleagues	1	583	57.85	58.33	21.88	0.91
Social support from colleagues	2	583	56.99	58.33	20.10	0.83
Social support from supervisor	1	584	72.77	75.00	19.98	0.83
Social support from supervisor	2	584	70.92	75.00	19.69	0.81
Social community at work	1	572	65.43	66.67	25.30	1.06
Social community at work	2	572	61.36	66.67	24.73	1.03
Job insecurity	1	587	25.72	25.00	21.27	0.88
Job insecurity	2	587	26.94	25.00	21.90	0.90
Job satisfaction	1	566	62.78	66.70	16.90	0.71
Job satisfaction	2	566	63.55	66.70	15.50	0.65
Work-family conflict	1	592	34.67	33.30	25.77	1.06
Work-family conflict	2	592	24.19	23.20	17.24	0.71
Family-work conflict	1	587	8.71	0.00	17.88	0.74
Family-work conflict	2	587	9.25	0.00	16.93	0.70
Mutual trust between employees	1	585	59.31	58.33	20.07	0.83
Mutual trust between employees	2	585	59.09	58.33	19.07	0.79
Trust regarding management	1	589	65.87	62.50	19.18	0.79
Trust regarding management	2	589	64.60	62.50	17.74	0.73
Justice and respect	1	589	59.74	62.50	20.87	0.86
Justice and respect	2	589	60.35	62.50	18.85	0.78
Social inclusiveness	1	590	66.26	68.75	18.89	0.78
Social inclusiveness	2	590	63.73	62.50	19.23	0.79
Sleeping trouble	1	596	27.75	25.00	22.30	0.91
Sleeping trouble	2	596	28.78	25.00	21.91	0.90
Burnout	1	595	39.24	43.75	19.94	0.82
Burnout	2	595	38.07	37.50	19.34	0.79
Stress	1	594	33.90	31.25	19.20	0.79
Stress	2	594	33.15	31.25	18.67	0.77
Depressive symptoms	1	595	24.68	25.00	16.68	0.68
Depressive symptoms	2	595	25.18	25.00	16.64	0.68
Somatic stress symptoms	1	588	18.92	12.50	16.26	0.67
Somatic stress symptoms	2	588	20.47	18.75	16.67	0.69
Cognitive stress symptoms	1	593	23.58	25.00	16.68	0.69
Cognitive stress symptoms	2	593	24.78	25.00	17.34	0.71
Self-efficacy	1	587	60.28	61.13	20.15	0.83
Self-efficacy	2	587	62.07	61.13	19.03	0.79

Note. N = sample size; M = mean value; Me = median; SD = standard deviation; SE = standard error of the mean.

### Confirmatory factor analysis

In order to perform the CFA analysis on the model with 119 items and 33 scales, it was necessary to replace the missing data with an *expectation-maximization* (EM) algorithm. Missing data constituted only 1.8% of all data points. The analyses were carried out using the *lavaan* and *semTools* libraries in the R statistical environment. Confirmatory analysis was carried out using the extraction of factors, such as ML–*maximum likelihood*. The following standard fit parameters were computed: *Root Mean Square Error of Approximation* (RMSEA), *Standardized Root Mean Squared Residual* (SRMR), *Comparative Fit Index* (CFI) and *Tucker–Lewis Index* (TLI). Although there is no complete consensus among authors as to the criteria for a "good fit" of the model [38], most assume that CFI and TLI > .90, RMSEA < .06 and SMRM < .08 indicate that the model represents a good or very good fit to the data. According to more relaxed criteria, the acceptable values are: CFI and TLI > .85, RMSEA < .08 and SMRM < 0.10 [39–41]. Table 2 presents the indices of the model fit to the data.

**Table 2:** Model adequacy and goodness-of-fit indexes of the model.

	*Χ* ^*2*^	*df*	*p*	*Χ* ^*2*^ */df*	RMSEA	SRMR	CFI	TLI	AIC
Model									
	13994.83	6374	< .001	2.20	.045	.06	.81	.79	626661.68

*Note*. RMSEA = root mean square error of approximation; SRMR = standardized root mean square residual; CFI = comparative fit index; TLI = Tucker Lewis index; AIC = Akaike’s information criterion; No respecifications of model were used.

Despite the restrictive assumptions of the confirmatory analysis, the model showed a good fit. Although the *χ*^*2*^ statistic proved to be statistically significant–a typical result for large sample sizes–all other parameters indicated acceptable or good fit. The *CMIN* (*χ*^*2*^*/df*) coefficient showed almost ideal fit values (concentrating around 2), RMSEA was below .05, and SRMR was below .08, which indicates a very good fit. The CFI and TLI values were slightly lower than acceptable but considering that they were obtained without the necessity of additional "interfering" with the model by means of modification indexes–e.g. imposing measurement error covariance or removing items from subscales–they do not seem to be that problematic.

Further, the factor loadings for each item were analyzed. According to one standard, acceptable factor loadings should be greater than .32 [42]. All factor pathways proved to be significant, which indicates a good fit of the model. The values of factor loadings were mostly high or very high and ranged from .29 to .95 for individual subscales. Although the value of .29 may seem relatively low, given the complexity of the model and the number of analyzed parameters, it can be assumed that it is an acceptable value. Table 3 presents factor loadings for all subscales included in the model. In all tables, factor numbers reflect the subscale numbers of the original COPSOQ II. Subscale no 27 (General Health) was excluded from all analyses since it consisted of only one item.

**Table 3:** Factor loadings/regression weights and significance of the paths for the subscales of COPSOQ II for the first measurement point, N = 599, N_items_ = 119.

	Coefficient	*SE*	*z*	*p*	Factor loadings
F1 – Quantitative demands					
QD1	17.19	1.06	16.27	< .001	.70
QD2	12.92	1.30	9.95	< .001	.58
QD3	16.10	1.19	13.58	< .001	.65
QD4	11.65	1.32	8.80	< .001	.48
F2 – Work pace					
WP1	16.51	1.01	16.31	< .001	.71
WP2	16.61	1.05	15.79	< .001	.70
WP3	16.65	0.96	17.34	< .001	.74
F3 – Cognitive demands					
CD1	13.39	1.22	10.98	< .001	.55
CD2	15.98	1.06	15.09	< .001	.60
CD3	17.95	0.95	18.99	< .001	.71
CD4	13.15	1.10	11.93	< .001	.55
F4 – Emotional demands					
ED1	17.16	1.03	16.61	< .001	.68
ED2	17.31	1.19	14.54	< .001	.63
ED3	17.74	1.02	17.38	< .001	.74
ED4	11.25	1.18	9.51	< .001	.53
F5 – Demands for hiding emotions					
HE1	13.82	2.15	6.42	< .001	.42
HE2	16.47	1.48	11.14	< .001	.59
HE3	13.48	1.83	7.35	< .001	.48
F6 – Influence					
IN1	14.31	1.69	8.47	< .001	.61
IN2	7.93	1.84	4.31	< .001	.29
IN3	16.82	1.40	12.01	< .001	.67
IN4	13.07	1.68	7.80	< .001	.49
F7 – Possibilities for development					
PD1	9.61	1.59	6.05	< .001	.43
PD2	17.60	1.25	14.07	< .001	.73
PD3	20.25	1.28	15.84	< .001	.76
PD4	20.92	1.11	18.91	< .001	.81
F8 – Variation					
VA1	23.72	3.18	7.46	< .001	.95
VA2	8.03	1.78	4.50	< .001	.30
F9 – Meaning of work					
MW1	11.60	1.59	7.29	< .001	.58
MW2	15.02	1.52	9.87	< .001	.65
MW3	15.50	1.28	12.08	< .001	.64
F10 – Commitment to the workplace					
CW1	10.94	1.48	7.38	< .001	.41
CW2	18.30	1.29	14.15	< .001	.63
CW3	11.87	1.50	7.91	< .001	.44
CW4	12.80	1.24	10.34	< .001	.60
F11 – Predictability					
PR1	16.65	0.98	16.97	< .001	.67
PR2	16.31	0.96	17.01	< .001	.76
F12 – Rewards					
RE1	19.40	0.97	20.07	< .001	.77
RE2	21.12	0.89	23.64	< .001	.84
RE3	19.27	0.88	21.83	< .001	.81
F13 – Role clarity					
CL1	14.02	0.99	14.11	< .001	.65
CL2	14.86	0.88	16.95	< .001	.77
CL3	14.58	0.85	17.14	< .001	.77
F14 – Role conflicts					
CO1	18.76	1.11	16.86	< .001	.70
CO2	14.30	1.35	10.56	< .001	.51
CO3	19.97	1.13	17.73	< .001	.74
CO4	16.62	1.11	14.96	< .001	.63
F15 – Quality of leadership					
QL1	23.16	0.85	27.40	< .001	.88
QL2	24.40	0.81	30.25	< .001	.89
QL3	22.71	0.92	24.67	< .001	.89
QL4	24.00	0.88	27.24	< .001	.87
F16 – Social support from colleagues					
SC1	19.05	0.98	19.50	< .001	.78
SC2	21.47	0.96	22.44	< .001	.82
SC3	18.02	0.97	18.64	< .001	.69
F17 – Social support from supervisor					
SS1	24.20	1.04	23.19	< .001	.85
SS2	25.98	0.89	29.13	< .001	.94
SS3	21.07	1.00	21.10	< .001	.76
F18 – Social community at work					
SW1	17.83	0.89	19.94	< .001	.84
SW2	17.74	0.90	19.69	< .001	.81
SW3	19.38	0.93	20.91	< .001	.81
F19 – Job insecurity					
JI1	18.20	1.21	15.00	< .001	.65
JI2	19.59	1.16	16.83	< .001	.78
JI3	19.09	1.14	16.78	< .001	.71
JI4	14.81	1.38	10.73	< .001	.46
F20 – Job satisfaction					
JS1	15.86	1.10	14.38	< .001	.71
JS2	16.18	1.06	15.21	< .001	.74
JS3	13.90	1.03	13.51	< .001	.73
JS4	14.83	1.05	14.12	< .001	.78
F21 – Work-family conflict					
WF1	14.68	1.38	10.65	< .001	.46
WF2	26.84	0.99	27.18	< .001	.86
WF3	27.65	1.01	27.29	< .001	.91
WF4	24.67	1.21	20.41	< .001	.73
F22 – Family-work conflict					
FW1	16.98	1.44	11.83	< .001	.88
FW2	15.31	1.29	11.92	< .001	.90
F23 – Mutual trust between employees					
TE1	23.24	1.05	22.15	< .001	.88
TE2	21.02	1.09	19.25	< .001	.79
TE3	8.82	1.41	6.24	< .001	.41
F24 – Trust regarding management					
TM1	17.04	1.02	16.73	< .001	.74
TM2	17.79	0.98	18.23	< .001	.77
TM3	12.00	1.36	8.79	< .001	.43
TM4	20.16	0.87	23.06	< .001	.80
F25 – Justice and respect					
JU1	20.73	0.89	23.39	< .001	.83
JU2	19.02	0.94	20.23	< .001	.73
JU3	19.07	0.83	23.02	< .001	.83
JU4	18.20	0.91	20.04	< .001	.77
F26 – Social inclusiveness					
SI1	15.97	1.21	13.19	< .001	.60
SI2	15.79	1.42	11.08	< .001	.57
SI3	14.42	1.11	12.94	< .001	.67
SI4	15.63	1.29	12.11	< .001	.57
F28 – Sleeping trouble					
SL1	21.50	0.88	24.47	< .001	.81
SL2	20.15	1.06	18.93	< .001	.77
SL3	20.75	0.94	22.11	< .001	.78
SL4	20.89	0.90	23.22	< .001	.85
F29 – Burnout					
BO1	18.47	0.72	25.55	< .001	.83
BO2	19.34	0.77	25.19	< .001	.83
BO3	18.22	0.79	23.19	< .001	.77
BO4	19.25	0.80	24.11	< .001	.83
F30 – Stress					
ST1	16.72	0.93	17.93	< .001	.66
ST2	16.25	0.81	19.95	< .001	.74
ST3	17.70	0.83	21.44	< .001	.77
ST4	19.00	0.81	23.56	< .001	.79
F31 – Depressive symptoms					
DS1	13.07	0.91	14.31	< .001	.60
DS2	16.36	0.82	19.98	< .001	.72
DS3	14.62	0.95	15.43	< .001	.64
DS4	12.92	0.82	15.69	< .001	.62
F32 – Somatic stress symptoms					
SO1	11.29	1.02	11.12	< .001	.54
SO2	14.73	0.99	14.84	< .001	.62
SO3	12.45	0.90	13.78	< .001	.62
SO4	16.22	0.84	19.39	< .001	.70
F33 – Cognitive stress symptoms					
CS1	15.48	0.77	20.05	< .001	.73
CS2	14.40	0.67	21.43	< .001	.72
CS3	13.91	0.87	16.06	< .001	.64
CS4	14.85	0.82	18.02	< .001	.67
F34 – Self-efficacy					
SE1	17.61	0.85	20.83	< .001	.73
SE2	17.70	1.02	17.39	< .001	.64
SE3	16.90	0.86	19.61	< .001	.72
SE4	19.61	0.84	23.46	< .001	.79
SE5	19.45	0.89	21.77	< .001	.77
SE6	19.87	0.88	22.60	< .001	.78

*Note*. Factor F27 (General Health) was excluded from analysis since it was consisting out of one item. Coefficient = unstandardized factor loading/regression coefficient. Std. all = standardized regression coefficients. The Item abbreviation is an accordance with the original COPSOQII.

### Reliability analysis

Next, we assessed the reliability of each subscale. The measures of the internal consistency analysis were Cronbach’s Alpha (α) and three coefficients Omega (ω), according to Raykov, Bentler and McDonald. According to Nunnally’s criterion, an internal consistency ratio above .60 is considered acceptable in some cases, above .70 is considered good and above .80 is considered very good [43]. Table 4 presents the reliability coefficients. The results of the analysis indicate that the vast majority of the COPSOQ II subscales demonstrate acceptable, good or very good measures of reliability. Four subscales have proved problematic in both measurements: variation, influence and commitment to the workplace, as well as demands for hiding emotions. Their measures of internal consistency yield below acceptable values; therefore, these subscales should be used with caution.

**Table 4:** Reliability coefficients for COPSOQ II subscales, N = 599.

Subscale	Cronbach’s alpha	Raykov’s omega	Bentler’s omega 2	McDonald’s omega 3
Quantitative demands	.70	.71	.71	.71
Work pace	.76	.76	.76	.76
Cognitive demands	.69	.70	.70	.69
Emotional demands	.73	.74	.74	.75
Demands for hiding emotions	.52	.53	.53	.53
Influence	.59	.58	.58	.56
Possibilities for development	.77	.79	.79	.81
Variation	.43	.60	.60	.60
Meaning of work	.65	.69	.69	.71
Commitment to the workplace	.59	.59	.59	.58
Predictability	.67	.67	.67	.67
Rewards	.85	.85	.85	.85
Role clarity	.76	.77	.77	.78
Role conflicts	.73	.74	.74	.75
Quality of leadership	.93	.93	.93	.93
Social support from colleagues	.79	.80	.80	.80
Social support from supervisor	.85	.85	.85	.85
Social community at work	.88	.89	.89	.89
Job insecurity	.73	.73	.73	.72
Job satisfaction	.82	.82	.82	.81
Work-family conflict	.82	.83	.83	.84
Family-work conflict	.88	.88	.88	.89
Mutual trust between employess	.71	.78	.78	.79
Trust regarding management	.76	.77	.77	.76
Justice and respect	.86	.86	.86	.86
Social inclusiveness	.69	.69	.69	.69
Sleeping trouble	.88	.88	.88	.88
Burnout	.89	.89	.89	.89
Stress	.82	.82	.82	.83
Depressive symptoms	.74	.74	.74	.74
Somatic stress symptoms	.71	.72	.72	.72
Cognitive stress symptoms	.78	.78	.78	.79
Self-efficacy	.88	.88	.88	.88

Although the model fit was acceptable, we have found some discriminatory problems with several subscales. Table 1A in the Appendix presents Pearson’s *r* correlational coefficients for the examined subscales/factors. In eight cases, the correlational coefficient exceeded the standard criterion of *r* = .85, which might indicate discriminatory problems and, thus, excessive similarities among some subscales. Cognitive demands (F3) and Emotional demands (F4) had a correlational coefficient value of *r* = .98; Meaning of work (F9) and Commitment to the workplace (F10), *r* = .92; Rewards (F12) and Trust regarding management (F24), *r* = .86; Quality of leadership (F15) and Social support from supervisor (F17), *r* = .86; Trust regarding management (F24) and Justice and respect (F25), *r* = 1.00 (especially problematic); Stress (F30) and Depressive symptoms (F31), *r* = .89; Stress (F30) and Cognitive stress symptoms (F33), *r* = .90; Depressive symptoms (F31) and Cognitive stress symptoms (F33), *r* = .98.

Generally, the results obtained indicate a good fit of the model to the data, despite its complexity and the discriminatory problems of several subscales. In all likelihood, the removal of questions with low factorial loadings from the questionnaire and, especially, the modification of the model based on modification indexes (e.g. imposition of the measurement errors covariance), would allow for a better fit. These measures, however, were decided against since the model already had an acceptable fit. Also, interference in the structure of the tool would make it difficult to compare the results of the survey using the Polish version of the questionnaire with the results obtained in other countries. In addition, the parameters of fit obtained in the study are satisfactory enough to confirm the structure of the tool in the version postulated by its authors [19].

The results of the test-retest reliability analysis are presented in Table 5. All observed *r*-Pearson correlation coefficients between the measured variables in the first and second measurements turned out to be positive and statistically significant, at the level of *p* < .001. The value of the correlation coefficients, however, is not high and ranges from *r* = .15 to *r* = .34. A broader reference to these results can be found in the discussion.

**Table 5:** Test-retest Pearson’s r reliability coefficients for two measurements.

Subscale	*r*	*p*
Quantitative demands	.29	< .001
Work pace	.28	< .001
Cognitive demands	.26	< .001
Emotional demands	.32	< .001
Demands for hiding emotions	.19	< .001
Influence	.22	< .001
Possibilities for development	.24	< .001
Variation	.21	< .001
Meaning of work	.22	< .001
Commitment to the workplace	.27	< .001
Predictability	.16	< .001
Rewards	.15	< .001
Role clarity	.17	< .001
Role conflicts	.33	< .001
Quality of leadership	.19	< .001
Social support from colleagues	.24	< .001
Social support from supervisor	.19	< .001
Social community at work	.19	< .001
Job insecurity	.34	< .001
Job satisfaction	.23	< .001
Work-family conflict	.24	< .001
Family-work conflict	.26	< .001
Mutual trust between employees	.26	< .001
Trust regarding management	.23	< .001
Justice and respect	.25	< .001
Social inclusiveness	.21	< .001
Sleeping trouble	.24	< .001
Burnout	.27	< .001
Stress	.25	< .001
Depressive symptoms	.32	< .001
Somatic stress symptoms	.21	< .001
Cognitive stress symptoms	.28	< .001
Self-efficacy	.20	< .001

*Note*. * *p* < .05,

** *p* < .01,

*** *p* < .001.

### Convergence validity

Table 6 presents the results of the correlation analysis for the convergence validity of the COPSOQ II Polish version. In general, high levels of five types of *demands at work* (measured with the COPSOQ II) were related to high quantitative workload (from *r* = .29 for quantitative demands to *r* = .56 for cognitive demands), work-family conflict (from *r* = .14 for cognitive demands to *r* = .45 for quantitative demands) and exhaustion (except cognitive demands, from r = .12 for emotional demands to *r* = .50 for quantitative demands). *Work organization and job contents* variables correlated positively with job satisfaction (from *r* = .21 for variation to *r* = .62 for commitment to the workplace) and negatively with disengagement from work (from *r* = –.27 for influence to *r* = –.60 for commitment to the workplace). *Interpersonal relations and leadership* variables were associated negatively with interpersonal conflicts at work (except predictability, from *r* = –.13 for social support from colleagues to *r* = –.36 for social community at work), and positively with high job satisfaction (from r = .28 for social support from colleagues to *r* = .48 for rewards), supervisor support (from *r* = .34 for role conflicts to r = .60 for quality of leadership) and coworker support (*r* = .29 for predictability to *r* = .53 for social community at work). *Work-Individual interface* variables were associated with high work-family conflict (from *r* = .14 for job insecurity to *r* = .63 for work-family conflict) and family-work conflict (from *r* = –.18 for job satisfaction to *r* = .42 for family-work conflict), as well as with low job satisfaction (except job insecurity, from *r* = –.19 for family-work conflict to *r* = .46 for job satisfaction). *Values at workplace* variables were positively related to job satisfaction (from *r* = .21 for mutual trust between employees to *r* = .45 for trust regarding management) and negatively related to exhaustion (from r = –.24 for social inclusiveness to *r* = –.39 for trust regarding management) and disengagement from work (from *r* = –.25 for mutual trust between employees to *r* = –.51 for trust regarding management). *Health and well-being* variables correlated with high level of depression (from *r* = .31 for burnout to *r* = .51 for depressive symptoms) and exhaustion (from *r* = .34 for sleeping problems to *r* = .48 for stress). The results of the analysis have confirmed the high theoretical accuracy of the Polish version of COPSOQ II.

**Table 6:** Pearson’s correlation coefficients for the COPSOQ II subscales and criteria variables, N = 599.

	Criteria Variables									
COPSOQ II Subscales	Quantitative workload	Interpersonal conflict at work	Work-family conflict	Family-work conflict	Depression	Exhaustion	Disengagement from work	Job satisfaction	Supervisors support	Coworkers support
**Quantitative demands**	.29***	.23***	.45***	.35***	.35***	.50***	.28***	-.22***	-.24***	-.26***
**Work pace**	.56***	.02	.25***	.12**	.07	.13**	-.02	-.02	.01	-.02
**Cognitive demands**	.46***	-.13**	.14**	-.03	-.07	-.02	-.20***	.08*	.14***	.15***
**Emotional demands**	.40***	-.09*	.21***	.01	.01	.12**	-.16***	.09*	.07	.08
**Demands for hiding emotions**	.37***	.02	.28***	.13**	.14**	.23***	.14*	-.12*	-.19**	-.13*
**Influence**	.10*	-.08	-.06	.03	-.06	-.21***	-.27***	.26***	.29***	.25***
**Possibilities for development**	.24***	-.13**	.02	.01	-.10*	-.23***	-.47***	.42***	.32***	.27***
**Variation**	.16***	-.03	.03	.04	-.03	-.11*	-.26***	.21***	.17***	.16***
**Meaning of work**	.27***	-.22***	-.09*	-.14**	-.18***	-.28***	-.46***	.42***	.32***	.25***
**Commitment to the workplace**	.02	-.09*	-.18***	-.09*	-.12	-.44***	-.60***	.62***	.36***	.24***
**Predictability**	-.05	-.07	-.15***	-.09*	-.16***	-.32***	-.38***	.34***	.39***	.29***
**Rewards**	.01	-.22***	-.14**	-.08	-.15***	-.35***	-.50***	.48***	.54***	.25***
**Role clarity**	-.16***	-.34***	-.14**	-.26**	-.27***	-.29***	-.37***	.34***	.35***	.31***
**Role conflicts**	.24***	.28***	.32***	.31***	.38***	.38***	.37***	-.28***	-.34***	-.31***
**Quality of leadership**	-.02	-.17***	-.23***	-.12**	-.22***	-.36***	-.46***	.43***	.60***	.31***
**Social support from colleagues**	-.01	-.13**	-.15***	-.09*	-.17***	-.24***	-.28***	.28***	.35***	.43***
**Social support from supervisor**	.03	-.17***	-.15***	-.12**	-.19***	-.29***	-.34***	.30***	.57***	.35***
**Social community at work**	.12**	-.36***	-.14**	-.23***	-.34***	-.31***	-.36***	.30***	.37***	.53***
**Job insecurity**	.08	.27***	.14**	.27***	.36***	.18***	.13**	-.07	-.20***	-.26***
**Job satisfaction**	-.04	-.17***	-.30***	-.18***	-.31***	-.37***	-.45***	.46***	.47***	.39***
**Work-family conflict**	.29***	.26***	.63***	.33***	.36***	.51***	.37***	-.33***	-.25***	-.22***
**Family-work conflict**	-.06	.36***	.25***	.42***	.38***	.30***	.28***	-.19***	-.16***	-.20***
**Mutual trust between employees**	-.21***	-.17***	-.24***	-.23***	-.28***	-.28***	-.25***	.21***	.28***	.36***
**Trust regarding management**	-.01	-.28***	-.27***	-.26***	-.27***	-.39***	-.51***	.45***	.60***	.31***
**Justice and respect**	-.07	-.12**	-.28***	-.18***	-.21***	-.38***	-.48***	.44***	.54***	.32***
**Social inclusiveness**	.15***	-.29***	-.12**	-.19***	-.21***	-.24***	-.34***	.26***	.38***	.36***
**Sleeping trouble**	.07	.23***	.18***	.10*	.33***	.34***	.21***	-.17***	-.21***	-.20***
**Burnout**	.16***	.19***	.31***	.13**	.31***	.43***	.23***	-.25***	-.18***	-.16***
**Stress**	.10*	.22***	.26***	.17***	.44***	.48***	.29***	-.29***	-.19***	-.20***
**Depressive symptoms**	-.06	.37***	.25***	.22***	.51***	.42***	.30***	-.29***	-.25***	-.28***
**Somatic stress symptoms**	-.05	.38***	.14**	.15***	.49***	.41***	.30***	-.25***	-.22***	-.24***
**Cognitive stress symptoms**	-.02	.36***	.28***	.23***	.47***	.45***	.31***	-.27***	-.24***	-.25***
**Self-efficacy**	.03	-.05	-.14**	-.12**	-.20***	-.25***	-.16***	.19***	.17***	.13**

Note. Criteria variables: quantitative workload, interpersonal conflicts at work, work-family conflict, family-work conflict, depression, exhaustion, disengagement from work, job satisfaction, supervisors support, coworkers support.

*Note*. * *p* < .05,

** *p* < .01,

*** *p* < .001.

## Discussion

The aim of the paper was the full evaluation of the psychometric properties of COPSOQ II among social service professionals work in direct relationships with other people in Eastern part of Europe. The paper has examined the reliability measures, criteria accuracy and theoretical accuracy of the 33 COPSOQ II subscales. The conducted analyses have excluded single-item theoretical constructs. Reliability has been tested using two methods: internal consistency analysis and an absolute stability estimation method (or test-retest method). The analyses have confirmed the validity of internal consistency parameters (Cronbach’s alpha coefficients and three Omega coefficients) for the vast majority of subscales. Four subscales (demands for hiding emotions, variation, influence, and commitment to work), which yielded a reliability of less than .60, should be used with caution. Slightly poorer results have been obtained for the absolute method stability. The *r*-Pearson correlation coefficients for each subscale in the first and second measurements ranged from *r* = .15 to *r* = .34. All were statistically significant at the *p* < .001 level, although they cannot be considered high. The quite low correlation coefficients may be related to the length of the interval between measurements. The optimal time interval has not been determined, as it depends to a large extent on the characteristics of the subject of measurement, the number of test items and the specificity of the research sample; however, it is recommended that the interval between measurements should range from a few weeks to a few months [44]. In the present study, the adopted interval was about 12 months, so it was slightly longer than recommended. Regarding the accuracy of the criteria, the CFA has supported the 33-subscale design of the tool.

Importantly, the analyses of this study have been based on the model proposed by the authors of the original version of the questionnaire [19]. The single model analysis, including 119 observable variables and 33 latent variables, produced satisfactory or good individual COPSOQ II subscales matching results. Note that no other studies are known to have confirmed the original COPSOQ II design in a similar way. Moreover, no other studies, to our knowledge, have even attempted to verify the structure of COPSOQ II treated as a complete instrument (without peaking particular subscales). To some extent, this is understandable since COPSOQ II is a very extensive and multidimensional tool, and this type of analysis requires lots of effort and computational power. Conversely, without a doubt, each psychological instrument requires psychometric validation. Thus, we believe that our study is without precedence and delivers important knowledge on the psychometric properties of the COPSOQ, also confirming its structure.

In our study, the theoretical accuracy of COPSOQ II, measured by the correlation of ten-criterion variables, has been confirmed. For example, demands at work variables in COPSOQ II were highly correlated with both job stressors (quantitative workload and work-family conflict) and poor occupational health (exhaustion). Work organization and job contents variables were related to high job resources (supervisor and coworker support), while health and well-being variables were negatively associated with depression and job satisfaction. Notably, a sufficiently high and steady pattern of correlation results with variables relating to the same phenomena but measured with different measurement tools: depressive symptoms–depression, burnout–exhaustion/disengagement from work, colleagues’ support–co-workers’ support, has been observed. Conversely, the relationships of some variables measuring very similar constructs were not very high (e.g. quantitative demands–quantitative workload).

A limitation of the Polish COPSOQ database and the derived reference values, however, is that the data were collected from a relatively small population and are not based on a representative sample of the employed population. Therefore, the results may be limited to human service workers only. The overall conclusions are: (1) the first full validation confirmed the structure proposed by the authors of the tool; (2) COPSOQ II is characterized by good psychometric parameters and could be successfully used to fulfill the demand for good and comprehensive assessment methods, also in the Polish job market settings. The research outcomes may enable further international comparative studies, particularly in the Polish context, where there has been very limited availability of developed measurement methods accounting for the changing nature of the work environment, including working conditions, contemporary requirements of modern organizations, the specificity of the current labor market and new forms of work.

The present study has been conducted among workers of the so-called social mission professions. Recently political and economic changes in Poland have modified the proffessions. There was rapid professionalization and bureaucratization of human services institutions. From small local agencies, these gradually evolved into large, modern organizations with extensive bureaucratic structures [45]. This process was accompanied by weakening formalized relations between employees. The professional attitude of human service workers also changed from being vocation-based and exhibiting strong commitment to more business-based, profit-oriented services. For many employees, who had a sense of mission, these changes resulted in the weakening of the importance of their work and their role in it, a decrease in identification with their profession and institution, as well as a tendency to leave the profession. According to the latest data on Poland, these occupations have been affected by personnel shortages [46]. According to the report *Health at a Glance*. *Europe 2018* [47] prepared by the OECD, Poland has a lower number of employed nurses per 1,000 inhabitants than the average in the EU (5.2 compared to 11.1 in Sweden, 14.3 in Finland, 16.9 in Denmark and 17.5 in Norway). Similar disproportions occur in the case of practicing doctors per 1,000 citizens (2.4 in Poland compared to 4.3 in Sweden, 3.2 in Finland, 3.7 in Denmark and 4.5 in Norway). Regarding doctors, the problem has been caused by medical studies admission quotas, long career paths and qualified specialists undertaking employment abroad. The shortage of nurses and midwives has been due to the reluctance of young people to enter the profession, the lack of valid permissions of qualified employees returning to work after a career break, and the retirement of experienced workers.

## Supporting information

S1 Database (SAV)
